# Mandibular incisive canal-related prevalence, morphometric parameters, and implant placement implications: a multicenter study of 847 CBCT scans

**DOI:** 10.4317/medoral.23350

**Published:** 2020-03-06

**Authors:** Daniel Almeida Ferreira Barbosa, Lúcio Mitsuo Kurita, Alynne Vieira de Menezes Pimenta, Renata Cordeiro Teixeira, Paulo Goberlânio Barros Silva, Thyciana Rodrigues Ribeiro, Daniela Pita de Melo, Fábio Wildson Gurgel Costa

**Affiliations:** 1 DDS, MSc. Division of Oral and Maxillofacial Radiology, School of Dentistry, Federal University of Ceará, Fortaleza, Brazil; 2 DDS, PhD. Division of Oral and Maxillofacial Radiology, School of Dentistry, Federal University of Ceará, Fortaleza, Brazil; 3 DDS, PhD. Division of Oral and Maxillofacial Radiology, School of Dentistry, Fortaleza University (UNIFOR), Brazil; 4 DDS, PhD. Division of Clinical Dentistry, School of Dentistry, Federal University of Ceará, Fortaleza, Brazil; 5 DDS, PhD. Division of Oral and Maxillofacial Radiology, School of Dentistry, State University of Paraíba, Campina Grande, Brasil

## Abstract

**Background:**

This study evaluated the epidemiological and morphological features of the mandibular incisive canal (MIC) using cone beam computed tomography (CBCT) in a significant sample of subjects in Brazil.

**Material and Methods:**

This retrospective, multicenter study assessed 847 CBCT scans performed at four oral imaging centers. The sample comprised CBCT images acquired from dentate individuals who presented at least from tooth 35 to tooth 45 in the anterior mandible region. Data regarding patient sex and age, and MIC linear measurements (length and diameter in mm), anatomical distances (to the alveolar, buccal and lingual cortexes, inferior border of the mandible, and adjacent teeth apexes), and location were obtained.

**Results:**

The MIC was more prevalent in women (76.3% [*p*<0.001]) between the fourth and sixth decades of life (*p*<0.001). It was present bilaterally (*p*<0.001) and exhibited a mean length of 7.7 mm (standard deviation [SD]=3.7 mm). Spearman correlation and logistic regression analysis revealed collinearity between age and linear measurements (*p*<0.05). The mean distances varied from the initial to the final portion of the MIC, respectively, in relation to the buccal cortex (mean=2.6 mm, SD=1.27; mean=3.96 mm; SD=1.43), to lingual cortex (mean=5.13 mm; SD=1.7; mean=4.61 mm, SD = 1.65), and to the inferior mandibular border (mean = 9.32 mm, SD=1.92; mean=8.76 mm, SD=2.07 mm). The difference in the proximity of the MIC to the apex of the inferior lateral incisor was statistically significant (*p*<0.05).

**Conclusions:**

Results of this study revealed a high prevalence of MIC with a bilateral pattern in women who were between the fourth and sixth decades of life. Both the distance between the MIC and the lingual cortex of the mandibular alveolar bone, and the diameter of the MIC, decreased as its trajectory assumed a more anterior position.

** Key words:**Mandibular incisive canal, cone-beam computed tomography, mandible, cross-sectional studies, anatomy.

## Introduction

Surgical procedures performed in the anterior portion of the mandible have traditionally been considered to be safe (1). However, recent evidence does not support the designation of this region as a “safe zone” based on significant reports in the literature related to surgeries for implants that have described complications associated with mandibular incisive canal (MIC) laceration or damage, including excessive bleeding in the mouth floor (2), neurosensory disorders (3) after removal of bone grafts (e.g., lower lip hypaesthesia and altered pulp sensitivity of anterior mandibular teeth), persistent pain sensation during surgical procedure for implant placement (4), and bleeding immediate to implant osteotomy (5).

Although previous studies have generally neglected the occurrence of the MIC (6), ex-vivo evaluation of the human mandible has demonstrated that it is a significant finding (7). Currently, the MIC is described as a well-defined canal that serves as a conduit for the incisive nerve, one of the terminal branches of the inferior alveolar nerve (8).

The detection, trajectory, and dimensional assessment of the MIC using cone beam computed tomography (CBCT) are considered important aspects of planning surgical procedures performed in the mandibular anterior region (MAR) (4). The first study to compare linear anatomical measurements of the MIC using CBCT and direct measurements on dry human mandibles concluded that there was no significant difference between the methods (9).

Previous studies using CBCT have reported a high prevalence of the MIC (10,11), and have emphasized the significant variation in the morphology of this anatomical structure and other anatomical structures of the interforaminal region (12). These variations in MIC image presentation can be attributed to different study populations, image acquisition protocols, and even to small study samples (13). Therefore, this study aimed to assess the epidemiological and morphometric features of the MIC using CBCT in a large multicenter sample.

## Material and Methods

- Sample

The study sample included CBCT data from patients who were referred to and underwent CBCT at one of four oral imaging centers (two university centers and two private clinics) between January 2015 and August 2017. All CBCT scans were referred for different clinical purposes, and were evaluated according to inclusion and exclusion criteria by three investigators (RCT, TRR, and DPM). The inclusion criteria were as follows: age, 18 to 69 years; CBCT performed to visualize the MAR; and CBCT images acquired from dentate individuals (at least in the anterior mandible region, from tooth 35 to tooth 45). Data from duplicated examinations, CBCT scans that revealed pathology or fractures, as well as facial growth disorders and syndromes, any artifacts (dental implants, fixing plates and/or screws) or motion artifacts, and low-quality diagnostic images were excluded.

- Variables

The independent variables analyzed in the present study included: sex; age; visibility of the MIC; number of MICs; vertical and horizontal diameters, and linear measurements; and the distance from the MIC to teeth apexes.

- Image Acquisition

CBCT data were acquired using one of four types of scanners: CS 9000 3D (Carestream Dental Rochester, NY, USA); Gendex CB-500 (Gendex Dental Systems, PA, USA; i-CAT Next Generation (Imaging International Sciences, Hatfield, PA, USA); and 4) i-CAT Classic (Imaging International Sciences, PA, USA).

**Figure 1:**
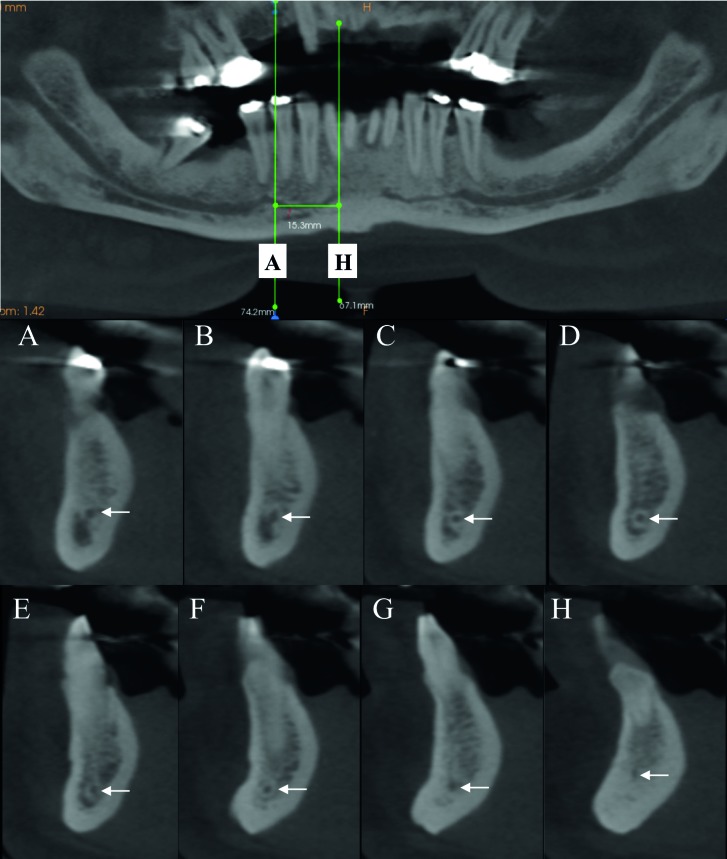
Representative figure of the adopted methodology. White arrows on the tomographic images indicate the mandibular incisive canal (MIC) from its initial portion (cross-sectional image #A) to its final portion (cross-sectional image #H). Reconstructed panoramic image indicating the length measurement of the MIC, which is represented by a line drawn from its origin to its final portion.

**Figure 2:**
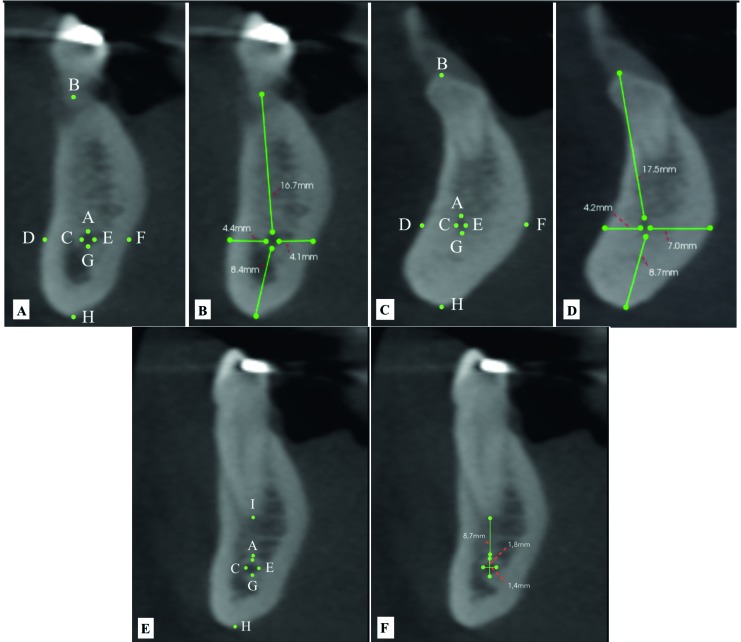
Distance from the mandibular incisive canal (MIC) to the alveolar bone crest (line drawn between points A and B), buccal plate (line drawn between points C and D), lingual plate (line drawn between points E and F), and inferior cortex (line drawn between points G and H). Distance from the MIC to adjacent teeth apexes (line drawn between points A and I). MIC length (line drawn from its origin to its final portion). MIC vertical (line drawn between points A and G) and horizontal (line drawn between points C and E) diameters.

All evaluations were performed by a trained observer (DAFB) in a dedicated room with dimmed light. All files were assessed using Carestream 3D Imaging Software (Carestream Dental Rochester, New York, USA). Initially, a panoramic reconstruction image using the inferior border of the mandible as a reference was obtained to guide the observer. Then, through cross-sectional images, the presence or absence of the MIC was determined. When the MIC was observed, its complete trajectory, the initial and final portions were identified to perform the following measurements (Fig. 1, Fig. 2).

1. Distance from the MIC to the alveolar bone crest (represented by a line drawn from a point designated as “B”, which was located at the MIC upper cortical, to another point designated as “A”, which was located at the uppermost point of the alveolar bone crest), buccal plate (represented by a line drawn from a point designated as “D”, which was the most buccal point of the MIC, to another point designated as “C”, which was located at the most external aspect of the buccal cortex), lingual plate (represented by a line drawn from a point designated as “F”, which was the most lingual point of the MIC, to another point designated as “E”, which was located at the most external aspect of the lingual cortex), and inferior cortex (represented by a line drawn from a point designated as “H”, which was located at the MIC inferior cortical, to another point designated as “G”, which was located at the most inferior point of the mandibular base);

2. Distance from the MIC to adjacent teeth apexes (represented by a line drawn from the point “A” to a point designed as “I”, which was located at the tooth apex);

3. MIC length (represented by a line drawn on the reconstructed panoramic image from its origin to its final visualization);

4. MIC vertical (represented by a line drawn from point “A” to point “G”) and horizontal diameter (represented by a line drawn from point “C” to point “E”).

To avoid any possible bias, an observer (DAFB) was trained and calibrated by the senior investigators (FWGC, LMK, and AVMP) with experience in oral and maxillofacial radiology to identify the presence or absence of the MIC and to perform the linear measurements described above. An image dataset of 30 CBCT scans was used. The same procedure was repeated within a 15-day interval. Data were assessed using IBM SPSS version 20.0 (IBM Corporation, Sommers, NY, USA) for Windows (Microsoft Corporation, Redmond, WA, USA).

To determine the sample size required to perform this study, the Cochran formula was used. The main variable for which the sample size was estimated was the number of individuals between 18 and 69 years of age [4,980,817], informed by an official national statistics agency (Brazilian Institute for Geography and Statistics; http://cod.ibge.gov.br/DAS). Additionally, a maximum sample error of 5% was adopted and a sample proportion was fixed at 0.5. Thus, a minimal sample of 384 CBCT scans was necessary to consider the sample to be representative.

- Statistical Analysis

To assess reproducibility, the following analyses were performed: Cohen’s kappa test for categorical data; intraclass correlation coefficient (ICC) to assess systematic errors related to numerical data; and the Dahlberg formula to assess casual errors from the linear measurements. The kappa coefficient reflects poor agreement [0], discreet agreement [0.01–0.2], relative agreement [0.21–0.4], moderate agreement [0.41–0.6], substantial agreement [0.61–0.8], and almost perfect agreement [0.81–1], according to Lands and Koch [1977]. To assess the ICC, a random bidirectional effect model with 95% confidence interval was used, with *p*<0.05 considered a satisfactory value.

The Kolmogorov-Smirnov test was used to test the normality of the data. The linear measurements are expressed as mean and standard deviation (SD), and categorical data are expressed in absolute and relative frequencies. Bivariate analysis was performed using the Wilcoxon (linear measurements versus side) and Mann-Whitney (linear measurements versus sex) tests, and analysis of variance (ANOVA) corrected using Bonferroni adjustment (MIC distances to the adjacent teeth apexes). Spearman correlation and multiple logistic regression were used to evaluate correlation and interdependence, respectively, between age and linear measurements. The Kruskal-Wallis/Dunn test was used to analyze the coefficient of variation (CV%). All analysis were performed by an investigator (PGBS) adopting a 95% confidence level.

## Results

- Reliability

In identifying the MIC, the intra-observer kappa value was 1.00 (i.e., almost perfect agreement). For MIC linear measurements, the reproducibility and confidence of the method were significant, varying from satisfactory (r = 0.772) to highly satisfactory (r = 0.998). Evaluating technical errors for linear measurements, all measurements were satisfactory and did not exceed 0.5 mm.

- Sample description

Three thousand five hundred twenty-one CBCT scans were obtained from the four oral imaging centers involved in the present study. After the inclusion and exclusion criteria were applied, 2674 CBCT scans were excluded from the final sample for the following exclusion criteria: 1) age under 18 years-old and over 69 years-old (n = 355); 2) the region of interest did not appear in the CBCT volume (n = 1912); 3) duplicity of CBCT scans (n = 45); 4) presence of lesion that interfered in the MIC morphology and trajectory (n = 91); 5) presence of artifacts that could impair MIC visualization (n =145); and 6) low quality images (n = 40). The final sample was composed of 847 CBCT scans (CS 9000 3D, n=34; Gendex CB-500, n = 202; i-CAT Next Generation; n = 302; i-CAT classic, n = 309).

- General characteristics

Of 847 CBCT scans, 646 (76.3%) revealed at least one visible MIC. All scanners yielded images in which the MIC was visible: CS 9000 3D (n = 20 [3.1%]); Gendex CB-500 (n = 166 [25.7%]); i-CAT Next Generation (n = 220 [34.1%]); and i-CAT Classic (n = 240 [37.2%]). There were significantly more females than males (*p* < 0.001) (Table 1).

**Table 1:**
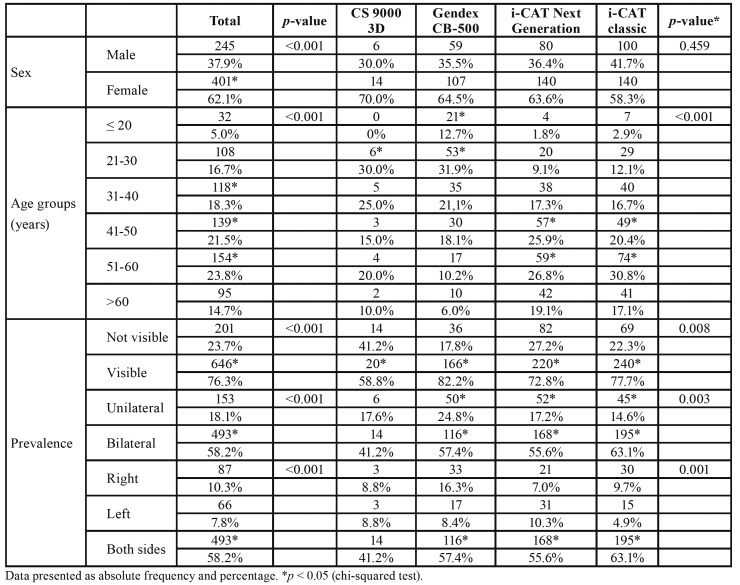
Sample characteristics according to sex, age group, and prevalence of a visible mandibular incisive canal (MIC) on cone beam computed tomography.

- Prevalence

The difference in presence of the MIC (76.3%) versus its absence (23.7%) was statistically significant (*p* < 0.001). Additionally, the difference between the number of examinations with bilateral presentation of the MIC (n = 493) and those with unilateral MIC (n = 153) was statistically significant (*p* < 0.001). However, there was no statistically significant difference in frequency of MIC occurrence on the left (7.8%) versus the right (10.3%) sides (*p* > 0.05).

- Linear measurements bivariate analysis

The mean length of the MIC 7.70 mm (SD = 3.70 mm), and did not differ between the left (mean = 8.09 mm, SD = 3.71 mm) and right (mean = 7.95 mm, SD = 3.78 mm) sides (*p* = 0.661). Linear measurements from the MIC to the alveolar cortex (mean = 16.63 mm; SD = 7.98 mm) and to the buccal cortex (mean = 2.60 mm; SD = 1.37 mm) for the right side of the mandible were significantly greater compared with the left side (Table 2).

MICs on the left side of the mandible exhibited a greater mean horizontal diameter at the final portion of the MIC (1.04 mm, SD = 0.36 mm; *p* = 0.005) and greater mean vertical diameter value at its initial portion (1.96 mm, SD = 0.38 mm; *p* < 0.001) than the right side (Table 2).

There were statistically significant differences when MIC length was compared between both sexes (Table 3). Males exhibited greater mean length values at its initial or final portions to the alveolar crest (17.23 mm and 19.55 mm, respectively), buccal cortex (2.75 mm and 4.32 mm, respectively), and inferior border of the mandible (9.98 mm and 9.60 mm, respectively) than females.

**Table 2:**
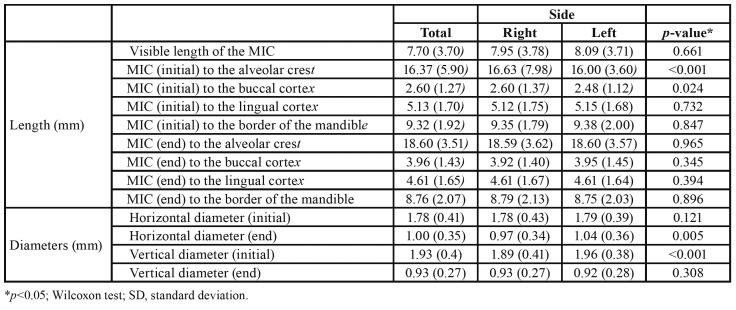
Bivariate analysis of side and linear measurements (length and diameters).

**Table 3:**
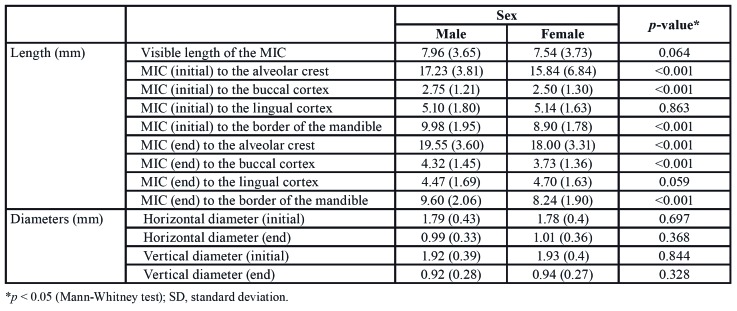
Bivariate analysis of sex and linear measurements (length and diameters).

**Figure 3:**
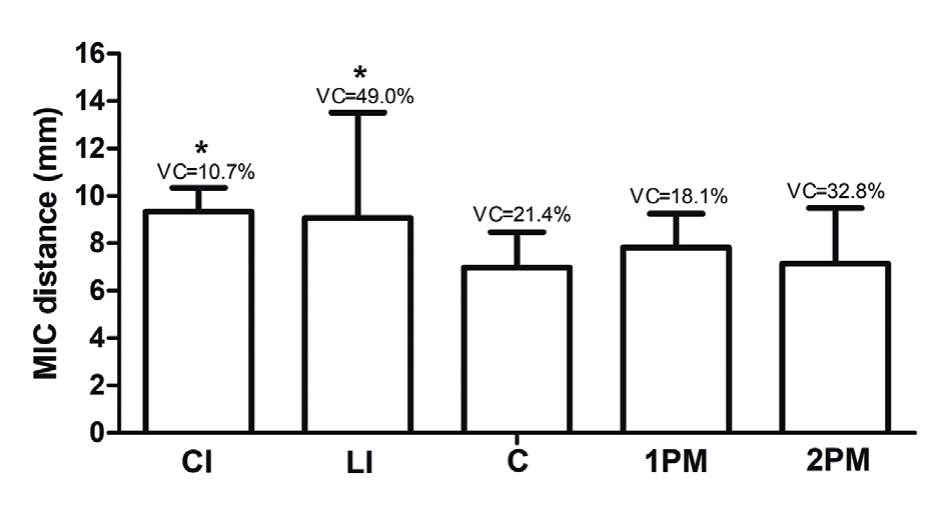
Distances from the apexes of the central incisors (CI), lateral incisors (LI), canines (C), first premolar (1PM), and second premolar (2PM) to the mandibular canal. *p < 0.05, Kruskal-Wallis/Dunn test (standard deviation [SD]). CV (%), coefficient of variation

Mean distances from the MIC to the adjacent teeth apexes ranged from 6.97 mm (SD = 1.58 mm) to 9.77 mm (SD = 1.04 mm), which were obtained at the canine and central incisor teeth apexes, respectively (Table 4). There was a statistically significant difference regarding the distance between the MIC and teeth apexes (*p* < 0.001). Fig. 3 illustrates the ratio of the SD to the mean, which represents the degree of variation (i.e., CV) of the tooth apex-MIC distance in the present study. Lateral incisors (CV 49%) and second premolars (CV 32.8%) exhibited a higher degree of variation in this measurement among teeth in the MAR, which was statistically significant (*p* < 0.05).

Age demonstrated a negative correlation with the distance between the initial portion of the MIC and the alveolar crest (*p* < 0.001; r = -0.392), the distance between the initial portion of the MIC and the buccal cortex (*p* = 0.001; r = -0.094), and the distance between the final portion of the MIC and the alveolar crest (*p* < 0.001; r = -0.312).

**Table 4:**
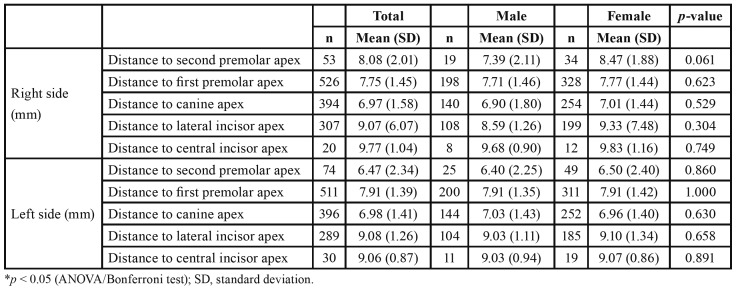
Bivariate analysis of sex and mandibular incisive canal (MIC) distance to the adjacent teeth apexes.

## Discussion

In this study, we performed an epidemiological and quantitative assessment of the MIC using data from 847 CBCT scans performed at different oral imaging centers and an appropriate methodology with adequate reproducibility. The anatomical structure assessed was localized in the MAR, which presents a fine alveolar process that usually requires attention during surgical procedures (14).

CBCT imaging results revealed a significant prevalence of MIC on at least one side of the mandible (76.3%). Previous studies have reported a MIC prevalence of 100% using CBCT imaging (15). Although Kong *et al*. (10) reported a 100% prevalence of MIC, the MIC was not clearly visible in 63.6% of the scans. Other studies have reported varied prevalence of the MIC, such as those by Apostolakis and Brown (15) (93%) and Parnia *et al*. (11) (83% with good visibility, 70.8% of MICs identified). Gomes *et al*. (16) found that 78% of MICs were visible among a sample population of 100 Brazilian patients. *Pi*res *et al*. (17) identified the MIC in 83.1% of evaluated CT scans, and 19.1% appeared unilaterally. In the present study, 18.1% of the MICs were unilateral canals, corroborating the results of a previous investigation (17).

Yang *et al*. (13) evaluated data from 411 CT scans, 246 of females (59.7%) and 166 of males (39.3%), which was in accordance with this study with regard to the high prevalence of MIC in women. Similarly, Pereira-Maciel *et al*. (18) reported that 63% of female patients exhibited a visible MIC. In the present study, the MIC was observed in 76.09% of females and 76.5% of males; as such, sex was not a statistically significant factor in relation to the presence of a MIC.

Gomes *et al*. (16) reported the greatest mean MIC length in the literature considering its male sample (22.6 mm). The mean length of the MIC according to measurements performed using the Gendex-CB 500 CBCT scanner in this study was 7.02 mm. In relation to investigations that used different image acquisition protocols, Apostolakis and Brown (15) reported a mean CIM length of 8.9 mm, while the present study found a mean length of 7.7 mm. However, this length is higher than the values for the right (7.1 mm) and left (6.6 mm) sides reported by *Pi*res *et al*. (18). In addition, previous studies have reported relatively greater mean lengths for the MIC, including 9.97 mm (13) and 12.4 mm (1).

The present study also analyzed morphometric variables and the side on which the MIC was visible, what are relatively scarce in the literature (10). Previous studies have reported similar lengths for both the left and right sides of the mandible: Apostolakis and Brown (9 mm and 8.8 mm) (15); and Kong *et al*. (17.73 mm and 17.84 mm) (10). This similarity between both sides of the mandible was also observed in this study (7.95 mm for the right side and 8.09 mm for the right side).

This study observed different mean horizontal diameters for the initial and final portions of the MIC (1.78 mm, SD 0.41 mm; and 1.00 mm, SD 0.35 mm, respectively). Kong *et al*. (10) reported values of 2.16 mm (SD 0.58 mm) and 0.84 (SD 0.23 mm), while the initial and final vertical diameters varied from 1.93 mm (SD 0.4 mm) to 0.93 mm (SD 0.27 mm), respectively. Kong *et al*. (10) reported mean values of 2.15 mm (SD 0.62 mm) and 0.89 SD (0.34 mm). Parnia *et al*. (12) reported mean diameters of 1.49 mm and 1.44 mm for the right and left sides, respectively. Orhan *et al*. (1) reported a mean value of 1.6 mm for the horizontal diameters and 1.2 mm for vertical diameters, but did not compare both sides. The present study found a mean vertical diameter of 1.96 mm for the right side and 1.89 mm for the left side in the initial portion of the MIC and 0.92 mm and 0.93 mm in the final portion.

The smaller diameter and poorer corticalization of the MIC compared with the mandibular canal make its visibility a challenge. In accordance with findings from the present study, the MIC exhibited an inferior and lingual path as it emerged from the mental foramen to the medium sagittal plane, with an increasing distance to the alveolar bone cortex, and assumed a buccal position. This trajectory is similar to the trajectory described by Mraiwa *et al*. (7).

This study revealed a significant difference in the distance between the initial portion of the MIC to the alveolar cortex, and this distance was larger for the right than the left side of the mandible. This finding appears to be attribuTable to the study population because it was from scans obtained from the three imaging centers with the largest proportion of CBCT scans in the entire study sample.

When correlating the distances assessed in this study with sex, there was a significant difference for the distance of the MIC to the alveolar cortex, buccal cortex, and inferior border of the mandible in males compared with females (*p* < 0.001). These findings corroborate those by *Pi*res *et al*. (17), who reported similar values for the initial and final portion of the MIC.

According to studies from the United States (17), Iran (20), China (10), and Brazil (18), the MIC was closer to the buccal cortex than to the lingual cortex during its intraosseous path, and in agreement with the present study. Lim *et al*. (19) reported that MIC deviated lingually from its starting point towards its endpoint in the mandible. Yang *et al*. (13) found that in the canine region, there was a decrease in the distance of the MIC to the lingual cortex. Angulated implants in this region may increase the risk for life-threatening hemorrhage because, in the canine region, the sublingual artery follows a horizontal course to the direction of the drill used during the surgical procedure, thus increasing the risk for laceration or transection (20). These findings, therefore, reinforce the necessity of CBCT imaging before surgical intervention in this region.

The MAR is not considered to be completely free of surgical complications (2,4). To collect a bone graft from the symphysis, a depth of 4 mm must be respected (21); the present study found a mean value of 3.96 mm similar to the value reported by Gomes *et al*. (3.9 mm) (16). When comparing our data with those in previous studies, our findings were smaller than the distance of 4.65 mm reported by Kong *et al*. (10). Yang *et al*. (13) recommended that implants in the interforaminal region should be inserted 10 mm above the inferior border of the mandible, which was also observed in the present study.

Negative collinearity between age and the distance between the MIC and the alveolar cortex is an interesting finding, which means that, as age increases, the referred distance tends to decrease. Clinically, this information affects the size of implant choice; therefore, a distance of 10 mm to the mandible inferior border is maintained (13). A negative correlation between age and MIC distance to the buccal cortex in its initial portion is in accordance with previous studies (15,21), which proposed a minimum distance of 3 mm as a safe distance for graft removal procedures.

The presence of negative collinearity between age and MIC distance to the alveolar cortex has not been previously reported and indicates that physiological bone resorption occurs in the alveolar region with increases in age. Therefore, this finding highlights the importance of the CBCT as an imaging modality of choice for routine examination when planning surgical procedures in the MAR (10).

The distance from the MIC to the adjacent teeth apexes through the MIC anterior course revealed that its distance to the left inferior second incisor was 9.08 mm (SD 1.26 mm) and right inferior second incisor, 9.07 mm (SD, 6.07 mm) and was significantly different from the canines and premolars. The studies by Yang *et al*. (13) and Kong *et al*. (10) reported similar values for MIC distances to teeth apexes (11.75 mm and 9.51 mm, respectively). Presently, it was an evident finding because the MIC was closer to adjacent teeth apexes in the posterior region.

Conclusion

In summary, the present study found a 76.3% prevalence of MIC visible in CBCT scans, which were obtained from four oral imaging centers. The presence of MIC was mainly related to subjects from 41 to 60 years of age, and this structure occurred frequently on both sides of the MAR. It exhibited a mean length of 7.7 mm, and different vertical and horizontal diameters in its initial and final portions. In addition, both the distance between the MIC and the lingual cortical of the mandibular alveolar bone, as well as the MIC diameter, decreased as its trajectory assumed a more anterior position. Those findings reinforce the importance of a systematic and careful analysis of the MIC using CBCT before surgical procedures involving the MAR.
